# Recommendation Map for Type 2 Diabetes: A Protocol for a Living Systematic Review of Clinical Practice Guidelines

**DOI:** 10.1002/gin2.70049

**Published:** 2025-10-13

**Authors:** Qiyi Zhang, Shichun Wang, Kang An, Arnav Agarwal, Britta Tendal Jeppesen, Per Olav Vandvik, Thomas Agoritsas, Heath White, Jun Tang, Wanru Wang, Chuanfeng Tao, Yaolong Chen, Sheyu Li, Yinghui Jin

**Affiliations:** ^1^ Center for Evidence‐Based and Translational Medicine, Zhongnan Hospital of Wuhan University; Center for Evidence‐Based and Translational Medicine Wuhan University Wuhan China; ^2^ School of Public Health Wuhan University Wuhan China; ^3^ General Practice Ward/International Medical Center Ward, General Practice Medical Center, West China Hospital Sichuan University Chengdu China; ^4^ Department of Endocrinology and Metabolism, Laboratory of Diabetes and Metabolism Research, West China Hospital Sichuan University Chengdu China; ^5^ MAGIC China Centre, Cochrane China Centre, Chinese Evidence-based Medicine Centre, West China Hospital Sichuan University Chengdu China; ^6^ Health Promotion and Food Nutrition & Safety Key Laboratory of Sichuan Province Chengdu China; ^7^ Department of Health Research Methods, Evidence and Impact McMaster University Hamilton Ontario Canada; ^8^ Division of General Internal Medicine, Department of Medicine McMaster University Hamilton Ontario Canada; ^9^ MAGIC Evidence Ecosystem Foundation Oslo Norway; ^10^ School of Public Health and Preventive Medicine Monash University Melbourne Victoria Australia; ^11^ Department of Medicine Lovisenberg Diaconal Hospital Oslo Norway; ^12^ Division of General Internal Medicine, Division of Clinical Epidemiology University Hospitals of Geneva Geneva Switzerland; ^13^ Department of Endocrinology and Metabolism Zhongnan Hospital of Wuhan University Wuhan China; ^14^ Evidence‐based Medicine Center, School of Basic Medical Sciences Lanzhou University Lanzhou China; ^15^ WHO Collaborating Centre for Guideline Implementation and Knowledge Translation Lanzhou China; ^16^ Research Unit of Evidence‐Based Evaluation and Guidelines, Chinese Academy of Medical Sciences (2021RU017), School of Basic Medical Sciences Lanzhou University Lanzhou China; ^17^ Institute of Health Data Science Lanzhou University Lanzhou China

**Keywords:** clinical practice guidelines, diabetes mellitus type 2, living systematic review, protocol

## Abstract

**Introduction:**

The global burden of type 2 diabetes, emergence of numerous new therapeutics and rapidly evolving evidence calls for trustworthy and current clinical practice guidelines (CPGs). This living systematic review of national and international CPGs aims to iteratively summarize recommendations for the management of type 2 diabetes.

**Method and Analysis:**

We will search electronic databases, professional academic association websites and guideline repositories from 2010 to April 1 2024 for national, international, or professional society CPGs addressing the management of type 2 diabetes. Our initial search will be followed by quarterly literature searches. Paired reviewers will independently assess the quality of identified CPGs using AGREE‐II. Recommendations and their associated methodological quality will be summarized and made available via a user‐friendly interactive online platform.

**Discussion:**

This living systematic review of CPGs will enable clinicians, patients and system‐level decision‐makers to inform their decision‐making based on internationally available CPGs. It will also allow methodologists to identify gaps in evidence and recommendations to inform future directions in research and CPGs development.

**PROSPERO Registration Number:**

CRD42023466448 (https://www.crd.york.ac.uk/PROSPERO/).

## Summary

- This study presents a protocol for a living systematic review that will continuously and iteratively synthesize and evaluate clinical practice guidelines on the management of type 2 diabetes, with quarterly updates.
- This study will evaluate the methodological rigor and recommendation consistency of existing type 2 diabetes guidelines, with the aim of identifying potential gaps and areas for future guideline development and clinical research.
- The recommendations will ultimately be presented in a user‐friendly, visual recommendation map.
- To enhance clarity and reproducibility, we have established well‐defined and operationalized criteria for recommendation extraction, explicitly distinguishing between “formal” and “informal” recommendations. These criteria are designed to minimize subjective bias, maximize the inclusion of relevant guidelines and ensure consistency of results throughout the data extraction process.

## 1 Introduction

Diabetes, a serious chronic condition, profoundly impacts millions of individuals worldwide [1, 2, 3]. In 2021, the global prevalence of diabetes among adults aged 20–79 has been estimated at 10.5%, affecting approximately 536.6 million people. This number is projected to rise to 783.2 million by 2045 [4]. Patients with type 2 diabetes have an increased risk of cardiovascular and kidney disease, leading to reduced quality of life and shorter life expectancy [5, 6].

As the number of patients continues to increase and awareness of the risks grows, and intensive glycemic control fails to significantly reduce risks, researchers and clinicians have been increasingly moving away from a glucose centric paradigm [7, 8]. The priority now is to reduce cardiovascular and chronic kidney disease through new and effective treatment strategies [9]. While a systematic review shows consistent relative risk reductions for some drug treatments, absolute benefits and risks depend on each patient's individual risk profile [10]. Therefore, there is an increasing focus on tailoring diabetes medications to a patient's baseline risk of cardiac and renal complications. Clinical practice guidelines (CPGs) are statements that include recommendations intended to optimize patient care [11]. Recent studies and CPGs offer clear recommendations for diabetes management, helping to manage the condition more effectively through implementing individualized and targeted recommendations [10, 12].

However, methodological limitations in diabetes guidelines, including redundant content across recommendations, inconsistencies in therapeutic prioritization, divergent approaches to risk stratification and prognostic evaluation, variability in methodological rigor, and delayed update cycles, collectively pose significant challenges to evidence‐based decision‐making in diabetes management [13, 14]. A living systematic review can be defined as an ongoing review process that continuously integrates relevant new CPGs as they become available [15, 16]. A living systematic review will be particularly important in fields where CPGs are emerging rapidly, and new research may change policy or practice decisions [17]. Due to the potential large volume of CPGs for type 2 diabetes and significant evolvement in treatment concepts and strategies over time, we will use evidence map to characterize the state of the recommendations, identify key gaps, and inform future research [18, 19]. We aim to (1) systematically review existing type 2 diabetes CPGs, with regular updates; (2) appraise their methodological rigour and the consistency of their recommendations; and (3) identify potential gaps and areas that should be addressed in future CPGs and clinical research.

## 2 Methods and Design

### 2.1 Step 1: Initial Review

We will begin by conducting and publishing a comprehensive initial systematic review of CPGs. This initial review will establish a baseline for subsequent, continuous updates.

#### 2.1.1 Search Strategy

We will conduct a three‐pronged search to capture eligible CPGs published between January 1 2010 and April 1 2024. We will perform an electronic database search using PubMed, Embase, Web of Science, Wan Fang Database, CNKI Database, using a combination of keywords and Medical Subject Headings (MeSH) terms including “Diabetes Mellitus”, “Diabetes Mellitus, Type 2” and “guidelines” (Appendix Table A1). To ensure maximum yield from our search, we will combine this with a comprehensive search across major professional society websites, including those of the American Diabetes Association, the European Diabetes Association, and the International Diabetes Federation; and across major guideline websites and databases such as those maintained by the National Institute for Health and Care Excellence and the Scottish Intercollegiate Guidelines Network (Appendix Table A2).

#### 2.1.2 Eligibility Criteria and Exclusion Criteria

Eligible CPGs will provide recommendations regarding the management of adults with type 2 diabetes. English‐language CPGs will be prioritized where multiple languages are available, and the most recent version will be prioritized where multiple versions or updates are available. We will exclude editorial or correspondence articles summarizing organizational guidelines.

#### 2.1.3 Study Selection

After duplicates are removed, paired reviewers will independently screen identified records at title and abstract and full‐text screening levels. Reasons for exclusion will be reported at the full‐text screening stage. Disagreements in judgements at either stage will be resolved through discussion or consultation with a team of endocrinologists and methodologists.

#### 2.1.4 Data Collection

For each CPGs, we will collect general information (title, author, publishing institution or association, country of origin, year of publication, and update status) and recommendation statements. We designed and used an Excel structured data extraction form to ensure consistency of data abstraction. We anticipate some CPGs will contain clear and easily identifiable recommendations, while others will require careful inspection to identify relevant statements.

Meeting any one of the following four points is sufficient to identify CPGs with recommendations [20, 21]:

1. Recommendations arranged in bulleted or list format.
2. In sentences presenting recommendations, the word “Recommendations” should start with a capital “R” or be in bold font to emphasize that the following text is a recommendation.
3. Recommendations are directly stated as formal recommendations, informal recommendations, or good practice statements.
4. The level of evidence is appended to the concluding sentence, with the strength of the recommendation indicated.

For CPGs with informal recommendations, written in paragraphs and/or in a narrative style, we have developed the following criteria for extracting recommendations [20, 21]:

1. The sentence should specify the population and recommended action.
2. Use of verbs such as ‘suggest,’ ‘advise,’ ‘apply,’ ‘consider,’ ‘recommend,’ ‘provide,’ ‘prefer,’ ‘measure,’ and ‘discuss’ can be preceded by modal verbs like ‘may,’ ‘should,’ etc., or adverbs such as ‘strongly’, ‘weakly’ or ‘conditionally’.
3. The use of imperative mood.
4. Negative and passive voice constructions can also be considered.
5. A conclusive sentence indicating the recommended action.

**Figure A1:**
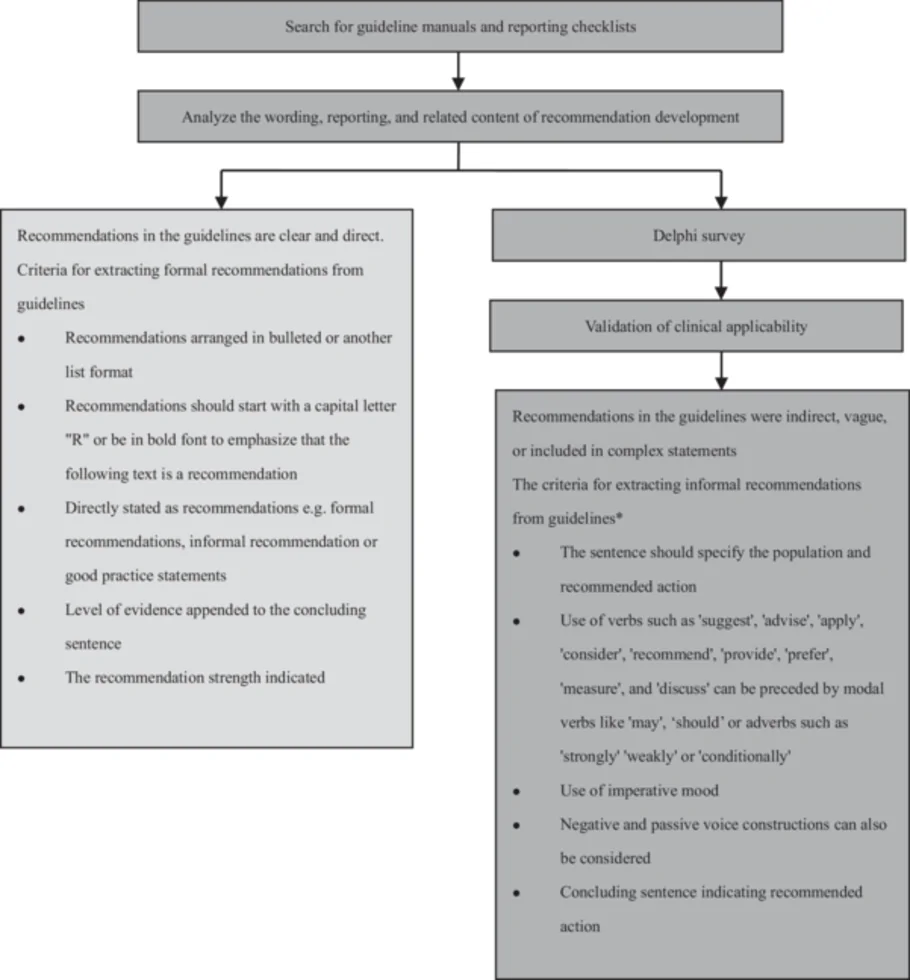
Generation processes of two criteria for identifying recommendations and extraction. *Process for developing criteria to extract informal recommendations from guidelines.

The process of generating these two sets of criteria is detailed in the appendix Figure A1. We will integrate the extracted guideline recommendations into a user‐friendly platform which present recommendations in a visual figure and searchable database. This integration aims to improve the efficiency of the research and analysis process and make diabetes guidelines and recommendations more accessible.

#### 2.1.5 Quality Appraisal

Paired reviewers will independently evaluate the methodological rigour of identified CPGs using the Appraisal of Guidelines for Research and Evaluation Instrument for Clinical Practice II (AGREE II). AGREE II comprises six domains – scope and purpose, stakeholder involvement, rigor of development, clarity of presentation, applicability and editorial independence – and 23 items [22, 23]. AGREE II has been selected for its capability to provide a thorough assessment of CPGs and its widespread acceptance as a tool for evaluating their robustness of development and transparency [24, 25, 26].

Two researchers will independently score each domain using AGREE II, and the intraclass correlation coefficient (ICC) will be calculated using SPSS 20.0 software to assess the consistency of the results among the two evaluators for each entry. Where the ICC is ≥ 0.80, it indicates good consistency. Where it is < 0.80, evaluators will undergo further training to discuss and independently assess all entries again. This process will continue until researchers show agreement on the assessment of every entry and the ICC reaches ≥ 0.80 [27]. Additionally, for each subsequent update of the systematic review, the ICC will be recalculated to ensure continued consistency and reliability in the evaluation process.

#### 2.1.6 Team Roles and Responsibilities

This study is conducted by a multidisciplinary team of clinicians (endocrinologists, internal medicine physicians and general practitioners) and methodologists with interest and expertise in type 2 diabetes management. Methodologists within the team have expertise with CPG development and appraisal. The review is also supported by an advisory team of endocrinologists who will provide clinical insights, address methodological queries, and resolve disagreements as they arise. To maintain transparency, any changes in team composition will be reported across review updates.

### 2.2 Step 2: Review Update

Our living systematic review will involve regular searches on a quarterly (every‐3‐month) basis. If new or updated CPGs are found, they will be incorporated into the analysis at time of update. If no new CPGs are found, the search date and associated results will be updated, and the analysis will remain unchanged.

Considering the advancements in diabetes research, we will continuously refine our search criteria and strategies in response to emerging recommendations. We aim to summarize and discuss the latest research findings, elucidate emerging recommendations, and clarify the significance of existing conclusions to ensure our review remains comprehensive and up‐to‐date. All modifications will be transparently documented in the open‐access protocol.

#### 2.2.1 Living Systematic Review Retirement

If sustained resources, both external and internal funding, are lacking or no new studies on the topic are expected to be published in the near future, specifically within the next 5 years, our team will divert efforts to other important research questions [28]. In such cases, a “RETIRED” label will be clearly displayed at the top of both the publicly accessible protocol page and the full text of the systematic review, accompanied by the retirement date and a brief explanation of the rationale behind the decision.

### 2.3 Step 3: Dissemination Strategy

We will use transparent open‐access methods to disseminate the results and updates of our living systematic review. We will create a ‘read‐only’ Microsoft Word document that can be accessed through the link provided in the comment posted in step one. The document will serve as a centralized repository for key resources, including:

1. Detailed Protocol
  The document will outline our review protocol, including any revisions made over time, ensuring transparency and accountability in our methodology.
2. Updated PRISMA Flow Chart
  We will provide an updated PRISMA flow chart detailing search date and returns from each iteration, offering insights into the review process and progress.
3. Living Data Extraction Sheet
  A comprehensive data extraction sheet will be maintained, featuring references for all included articles, allowing for transparency and reproducibility of our findings.
4. Key Findings Table
  This table will summarize the key findings from each iteration of the review, providing stakeholders with a concise overview of the evolving recommendations landscape.
5. Key Recommendations for Future Research
  Our document will include key recommendations for future research directions, identifying gaps and areas for further exploration in the field of diabetes guidelines.

By making these resources openly accessible, we aim to facilitate transparency, collaboration, and knowledge dissemination in the realm of diabetes guidelines.

## 3 Discussion

The treatment needs for diabetes are complex, covering various aspects including medication, dietary control, exercise therapy, and blood glucose monitoring [29, 30, 31]. CPGs help clinicians make more informed clinical decisions, enhancing treatment consistency and standardization to ensure patients receive optimal medical care [30, 32]. Our review will enhance evidence‐based decision‐making capabilities, serving as an essential resource for stakeholders seeking to comprehend the current landscape informed by the latest CPGs and recommendations.

This LRS will aim to comprehensively assess the quality of current CPGs, providing clinicians with precise treatment plans and management recommendations by presenting the recommendation map in a user ‐ friendly and visual format. The expected outcomes of this comprehensive evaluation include offering clinicians a practical guideline reference and supporting guideline developers in enhancing the quality of their CPGs, adapting CPGs and updating CPGs.

Moreover, we are committed to actively promoting the timely update of CPGs, ensuring that our efforts contribute to the ongoing improvement of healthcare practices. This effort seeks to seamlessly bridge the gap between clinical decision‐making and the implementation of CPGs.

## Author Contributions

**Qiyi Zhang:** Conceptualization and writing ‐original draft. **Shichun Wang:** Data curation and writing – review and editing. **Kang An:** Software; visualization and writing – review and editing. **Arnav Agarwal:** Methodology and writing – review and editing. **Britta Tendal Jeppesen:** Methodology and writing – review and editing. **Per Olav Vandvik:** Methodology and writing – review and editing. **Thomas Agoritsas:** Methodology and writing – review and editing. **Heath White:** Methodology and writing – review and editing. **Jun Tang:** Supervision and writing – review and editing. **Wanru Wang:** Data curation. **Chuanfeng Tao:** Data curation. **Yaolong Chen:** Methodology and writing – review and editing. **Sheyu Li:** Conceptualization; methodology; project administration and writing – review and editing. **Yinghui Jin:** Conceptualization; supervision; funding acquisition and writing – review and editing.

## Conflicts of Interest

The authors declare no conflicts of interest.

## Data Availability

No data are available as this is a protocol. Data sharing is not applicable to this article as no datasets have been generated or analyzed yet.

## 1

**Table A1:** Strategy of PubMed searching.

Search	Search term
#1	“Diabetes Mellitus”[Mesh] OR “Diabetes Mellitus, Type 2”[Mesh]
#2	“Type 2 Diabetes Mellitus”[Title] OR “Type II Diabetes Mellitus”[Title] OR “Type 2 Diabetes”[Title] OR “Type II Diabetes”[Title] OR “Noninsulin Dependent Diabetes Mellitus”[Title] OR “Adult‐Onset Diabetes Mellitus”[Title] OR “stable diabetes mellitus”[Title] OR “Maturity Onset Diabetes Mellitus”[Title] OR “Slow‐Onset Diabetes Mellitus”[Title] OR “Maturity Onset Diabetes”[Title] OR “ketosis resistant diabetes mellitus”[Title]
#3	#1 OR #2
#4	“Practice Guidelines as Topic”[Mesh] OR “Guidelines as Topic”[Mesh] OR “Practice guideline”[Publication Type] OR “Guideline”[Publication Type] OR “Consensus”[Mesh]
#5	“guideline*”[Title] OR “guidance*”[Title] OR “recommendation*”[Title] OR “CPG*”[Title] OR “Best Practice”[Title] OR “Clinical Practice Guideline”[Title] OR “Clinical Guidelines”[Title] OR “Expert consensus”[Title] OR “Standard*”[Title] OR “consensus statement”[Title]
#6	#4 OR #5
#7	#3 AND #6

**Table A2:** Search resources.

Resources	Website
Databases	
PubMed	https://pubmed.ncbi.nlm.nih.gov/
Embase	https://www.embase.com/
Web of science	https://www.webofscience.com/wos/
Scopus	https://www.scopus.com/home.uri
Wan fang data knowledge service platform	https://www.wanfangdata.com.cn/
China national knowledge infrastructure database	https://www.cnki.net/
China science and technology journal database	http://qikan.cqvip.com/
Academic associations	
American diabetes association	https://professional.diabetes.org/
European association for the study of diabetes	https://www.easd.org/
Canadian diabetes association	https://guidelines.diabetes.ca/home
American association of clinical endocrinologists	https://www.aace.com/
International diabetes federation	https://idf.org/
Italian society of diabetology	https://www.siditalia.it/
Japan diabetes society	https://www.jds.or.jp/
Polish diabetes association (polskie towarzystwo diabetologiczne)	https://www.ptdiab.pl/
Korean diabetes association	https://www.diabetes.or.kr/english/
New zealand society for the study of diabetes	https://www.t2dm.nzssd.org.nz/Home.html#subjects-list
Australian diabetes society	https://www.diabetessociety.com.au/
Malaysian endocrine and metabolic society	https://mems.my/
Research society for the study of diabetes in india	https://www.rssdi.in/newwebsite/page.php?id=114
Chinese diabetes society	https://diab.cma.org.cn/cn/index.aspx
Chinese society of endocrinology	https://endo.cma.org.cn/
Association of British clinical diabetologists	https://abcd.care/position-papers-and-guidelines
Joint British diabetes societies for inpatient care	https://abcd.care/jbds-ip
Canadian medical association	https://www.cma.ca/
American college of physicians	https://www.acponline.org/
U.S. preventive services task force	https://www.uspreventiveservicestaskforce.org/uspstf/
Institute for clinical systems improvement	https://www.icsi.org/guideline/
World health organization	https://www.who.int
American college of cardiology	https://www.acc.org/
European society of cardiology	https://www.escardio.org/
Australian cardiovascular alliance	https://ozheart.org/
Chinese society of cardiology	https://csc.cma.org.cn/
American society of nephrology	https://www.asn-online.org/
European renal association	https://www.era-online.org/
Kidney health Australia	https://kidney.org.au/
Chinese society of nephrology	https://csnchina.cma.org.cn/cn/
Kidney disease: improving global outcomes	https://kdigo.org/
Government of British Columbia	https://www2.gov.bc.ca/gov/content/home
Guideline website/databases	
National institute for health and clinical excellence	https://www.nice.org.uk/
Scottish intercollegiate guidelines network	https://www.sign.ac.uk/
National guideline clearinghouse	https://www.ahrq.gov/
Guideline international network	https://g-i-n.net/
Australian clinical practice guidelines	https://www.nhmrc.gov.au/guidelines

Some recommendations lack clarity, making it difficult for readers to understand and implement the guidelines effectively. This study aims to resolve this issue by developing criteria for identifying and extracting such informal recommendations from guidelines.

First, we searched guideline databases, professional society websites, organizational websites, and the EQUATOR Network to gather sufficient guideline manuals and reporting checklists to collect the requirements for recommendation wording and reporting. These materials were then analyzed to establish the initial criteria for extracting informal recommendations, which are as follows:

- The sentence should specify the target population and the recommended action.
- Verbs such as ‘suggest,’ ‘advise,’ ‘consider,’ ‘recommend,’ ‘measure,’ and ‘discuss’ may be preceded by modal verbs like ‘may,’ ‘should,’ or adverbs such as ‘strongly,’ ‘weakly,’ or ‘conditionally.’
- The use of negative and passive voice constructions can be considered when necessary.

Next, we applied the Delphi method to collect the opinions of guideline methodologists and clinicians from various levels of hospitals regarding the initial criteria. We summarized and analyzed the feedback, then repeated the process until a consensus was reached. After multiple rounds of surveys, participants generally agreed to add the following to the initial criteria:

- Supplementing recommendation verbs, including ‘apply,’ ‘prefer,’ and ‘provide.’
- Using the imperative mood to strengthen recommendations.
- Including a concluding sentence that clearly states the recommended action.

Finally, a simple random sampling method was used to select 30 guidelines containing informal recommendations. These recommendations were independently extracted by two researchers, verified by a third researcher, and subsequently evaluated by clinicians for clinical applicability. The results indicated that the recommendations extracted using our criteria were feasible and practical.

Our criteria provide a stable and reproducible method for extracting recommendations from informal recommendation guidelines, potentially reducing the challenges associated with implementing guidelines.
